# SELF-CARE AND QUALITY OF LIFE IN PEOPLE WITH PARKINSON’S DISEASE AND THEIR CAREGIVERS

**DOI:** 10.1093/geroni/igac059.2052

**Published:** 2022-12-20

**Authors:** Eunyoung Kim, JuHee Lee, Misook Chung, Subin Yoo

**Affiliations:** Yonsei University, Seoul, Seoul-t'ukpyolsi, Republic of Korea; Yonsei University, Seoul, Seoul-t'ukpyolsi, Republic of Korea; University of Kentucky, Lexington, Kentucky, United States; Yonsei University, Seoul, Seoul-t'ukpyolsi, Republic of Korea

## Abstract

**Objective:**

To examine associations of self-care and the quality of life (QOL) in patients with Parkinson's disease (PD) and their caregivers dyads.

**Background:**

Patients with PD engage in self-care with motor and non-motor symptom experiences. There is insufficient knowledge about associations of self-care and QOL in patients with PD and their caregivers though PD patients and family caregivers are interdependent. Method: A total of 73 PD patients and primary family caregivers from the Korean Parkinson's Disease Association or who visited outpatient clinics of the tertiary hospitals in Korea participated in this study. Dyad members completed the survey of the self-care (the Self-Care of Chronic Illness Inventory and Caregiver Contribution to Self-Care of Chronic Illness Inventory) and QOL (Parkinson’s Disease Quality of Life Questionnaire and The Parkinson’s Disease Questionnaire-Care). The comparisons of self-care and quality of life within two dyad members were conducted via paired t-test and Pearson's correlation using SPSS 26.0 Result: PD patients reported significantly higher levels of self-care maintenance (78.34 vs. 70.21, patients vs. caregiver respectively, p<.001) and management (65.9 vs. 60.49, p=.029) than caregivers. Patients’ self-efficacy was significantly correlated with their QOL (r=.270, p=.021). Caregiver contribution to self-care management was significantly correlated with their own QOL (r=.365, p=.001) and patients’ QOL (r=.234, p=.047).

**Conclusion:**

The self-care efficacy of patients and the contribution of caregivers to self-care management can affect the QOL of both patients and caregivers. A dyadic approach for the intervention of self-care management is crucial to improve the QOL of patients with PD and caregivers.

